# Estimation of divergence time between two sibling species of the *Anopheles (Kerteszia) cruzii *complex using a multilocus approach

**DOI:** 10.1186/1471-2148-10-91

**Published:** 2010-03-31

**Authors:** Luísa DP Rona, Carlos J Carvalho-Pinto, Camila J Mazzoni, Alexandre A Peixoto

**Affiliations:** 1 Laboratório de Biologia Molecular de Insetos, Instituto Oswaldo Cruz, FIOCRUZ, Av. Brasil 4365, Rio de Janeiro 21045-900, RJ, Brazil; 2 Departamento de Microbiologia e Parasitologia, CCB, Universidade Federal de Santa Catarina, Florianópolis 88040-970, SC, Brazil; 3 Environmental Research Institute, University College Cork, Cork, Ireland; 4 Current address: Evolutionary Genetics Group, Leibniz Institute for Zoo and Wildlife Research, Alfred-Kowalke-Str, 17, D-10315 Berlin, Germany

## Abstract

**Background:**

*Anopheles cruzii *is the primary human *Plasmodium *vector in southern and southeastern Brazil. The distribution of this mosquito follows the coast of the Brazilian Atlantic Forest. Previous studies indicated that *An. cruzii *is a complex of cryptic species.

**Results:**

A multilocus approach using six loci, three circadian clock genes and three encoding ribosomal proteins, was implemented to investigate in more detail the genetic differentiation between the *An. cruzii *populations from Santa Catarina (southern Brazil) and Bahia States (northeastern Brazil) that represent two sibling species. The analysis revealed very high *F*_*ST *_values and fixed differences between the two *An. cruzii *sibling species in all loci, irrespective of their function. An Isolation with Migration model was fit to the data using the IM program. The results reveal no migration in either direction and allowed a rough estimate of the divergence time between the two sibling species.

**Conclusions:**

Population genetics analysis of *An. cruzii *samples from two Brazilian localities using a multilocus approach confirmed that they represent two different sibling species in this complex. The results suggest that the two species have not exchanged migrants since their separation and that they possibly diverged between 1.1 and 3.6 million years ago, a period of intense climatic changes.

## Background

*Anopheles cruzii *(Diptera: Culicidae) is the primary vector of human and simian malaria parasites in southern and southeastern Brazil [1,2]. Earlier studies that evaluated *X *chromosome inversion frequencies [3,4] and isoenzyme profiles [5] suggest that *Anopheles cruzii *is a species complex. A recent analysis of genetic differentiation using the *timeless *gene among *An. cruzii *populations from southern, southeastern and northeastern Brazil indicated that the population from Itaparica, Bahia State (northeastern Brazil) is a different species [6].

In the current study, a multilocus analysis using six different nuclear gene fragments was performed comparing two populations of *An. cruzii *(Florianópolis and Itaparica), representing respectively the southeastern and northeastern sibling species. Three of the fragments used are orthologues of *Drosophila melanogaster *genes involved in the control of circadian rhythms: *timeless *(*tim*), *Clock *(*Clk*) and *cycle *(*cyc*); and three code for ribosomal proteins: *Rp49 *(Ribosomal protein 49, known also as RpL32 - Ribosomal protein L32), *RpS2 *(Ribosomal protein S2) and *RpS29 *(Ribosomal protein S29).

The aim of the study was to determine if there is still gene flow between the two sibling species and to estimate their divergence time. Furthermore, circadian genes [7] putatively involved in the control of mating rhythms [8], such as *timeless*, *Clock *and *cycle*, are potentially important in maintaining temporal reproductive isolation between closely related species. Based on that, this study also aimed to verify whether the differentiation in circadian genes is higher than the divergence in constitutive loci, such as the ribosomal protein genes *Rp49*, *RpS29 *and *RpS2*.

## Results

### Polymorphism and divergence between Florianópolis and Itaparica

One of the assumptions of the Isolation with Migration model used in this study is the absence of recombination within the studied loci. In order to fulfill this requirement, the optimal recombination-filtered block was extracted from each gene alignment (see below). Table 1 shows the position of the non-recombining (NR) blocks used in this study as well as the putative recombinant sequences that were removed (see Methods). Another assumption of the IM program is that the variation observed in the studied loci is neutral. Therefore, the Tajima [9] and Fu & Li [10] tests of neutrality were used and the results are presented in Table 2. No significant deviations from neutrality were observed after Bonferroni correction.

**Table 1:** NR blocks and sequences excluded from the IM analysis.

locus	NR blocks	Removed sequences
***timeless***	124 -- 381	Flo31a, Flo31b, Flo32b, Flo35a, Flo35b, Flo36b, Flo37a, Flo39a, Flo40a

***Clock***	1 -- 154	Bah02b, Bah03a, Bah03b, Flo08a, Flo12a, Flo16b

***cycle***	36 -- 131	Flo06a, Flo18a, Flo18b

***Rp49***	47 -- 269	Flo06a, Flo06b, Flo09b

***RpS2***	1 -- 266	Flo09b

***RpS29***	36 -- 274	Bah31b, Flo07b, Flo09b, Flo12a

Edition of sequences prior to IM analysis using the IM_GC _program and based on alignment presented in additional files 1, 2, 3, 4, 5 and 6. NR blocks, fragment positions of the non-recombining blocks used in the analyses; Removed sequences, the putative recombinant sequences removed before the IM analysis.

**Table 2:** Polymorphisms of *An. cruzii *sibling species from Florianópolis and Itaparica

Locus	Population	*RM*	*Length (bp)*	*n*	S	θ	π	*D*_*T*_	*D*_*FL*_	*F*_*FL*_
***timeless***	Florianópolis	14	413 (258)	24 (15)	59 (16)	0.04314 (0.02141)	0.03081 (0.02080)	-0.98320 (-0.02183)	-0.54978 (0.71094)	-0.80513 (0.58572)
	Itaparica			28 (28)	24 (14)	0.01661 (0.01602)	0.01035 (0.00994)	-1.31797 (-1.25698)	-0.83982 (-0.77081)	-1.16519 (-1.07989)

***Clock***	Florianópolis	03	159 (154)	24 (21)	10 (09)	0.01750 (0.01688)	0.01774 (0.01703)	0.11242 (0.09477)	-0.60737 (-0.19574)	-0.45842 (-0.12971)
	Itaparica			24 (21)	08 (06)	0.01592 (0.01282)	0.02253 (0.01789)	1.41474 (1.30746)	1.33358 (1.24418)	1.57772 (1.45949)

***cycle***	Florianópolis	05	218 (96)	24 (21)	21 (12)	0.02787 (0.03929)	0.02802 (0.02946)	-0.34121 (-0.77817)	0.56744 (0.44350)	0.50899 (0.25060)
	Itaparica			24 (24)	03 (03)	0.00370 (0.00845)	0.00304 (0.00691)	-0.43933 (-0.43933)	0.97946 (0.97946)	0.67147 (0.67147)

***Rp49***	Florianópolis	01	269 (223)	24 (21)	10 (09)	0.01134 (0.01299)	0.00849 (0.00876)	-0.82070 (-1.09222)	-0.43327 (-1.00506)	-0.63920 (-1.19531)
	Itaparica			24 (24)	09 (09)	0.00915 (0.01109)	0.00678 (0.00820)	-0.82379 (-0.82379)	-1.35698 (-1.35698)	-1.39541 (-1.39541)

***RpS2***	Florianópolis	01	270 (266)	24 (23)	17 (17)	0.01879 (0.01931)	0.01723 (0.01719)	-0.22879 (-0.33019)	0.94975 (0.95577)	0.69085 (0.66273)
	Itaparica			24 (24)	08 (07)	0.00807 (0.00716)	0.00863 (0.00679)	0.25353 (-0.13512)	0.73702 (0.62918)	0.69164 (0.47169)

***RpS29***	Florianópolis	02	274 (239)	24 (21)	13 (07)	0.01375 (0.00836)	0.00907 (0.00774)	-1.14756 (-0.21099)	-2.19895 (-0.60726)	-2.19619 (-0.57191)
	Itaparica			24 (23)	20 (13)	0.02101 (0.01499)	0.01002 (0.00877)	-1.87423 (-1.42007)	-2.78643 (-1.74321)	-2.93189 (-1.91842)

*RM*, the minimum number of recombination events; *n*, number of DNA sequences of each sibling species; *S*, number of polymorphic (segregating) sites; θ, nucleotide diversity based on the total number of mutation (*Eta*); π, nucleotide diversity based on the average number of pair-wise differences; *D*_*T*_, Tajima's D [9]; *D*_*FL*_, Fu & Li's D [10] and *F*_*FL*_, Fu & Li's F [10], based on *Eta *(total number of mutations). No significant deviations from neutrality were observed after Bonferroni correction. Numbers in parentheses are related to the non-recombining block (NR) for each locus.

Table 2 also shows the minimum number of recombination events for each gene (*RM*) and the length of the whole fragment and for the NR block of each gene (values in parentheses). The larger differences in length between the whole fragment and the NR block were observed for *timeless *and *cycle *and this was due to the higher number of recombination events identified in these two genes (*RM *= 14 and 5 respectively). The alignments of the whole sequences of each gene are presented in Additional files 1, 2, 3, 4, 5 and 6. All loci include at least one intron of variable size, except the *cycle *gene fragment, which was composed entirely of an exon. Except for the *timeless *gene, all base substitutions were synonymous or occurred within introns. The few non-synonymous changes found in the *timeless *gene are described in Rona *et al*. [6].

Table 2 also shows the number of polymorphic sites (S) for each *An. cruzii *sibling species and two measures of nucleotide diversity: π, based on the average number of pairwise differences and θ, based on the total number of mutations (values for the NR blocks in parentheses). In general, Itaparica was less polymorphic than Florianópolis, having showed the lowest θ and π values, as well as fewer polymorphic sites (S). Table 3 shows the pairwise estimates of population differentiation between the two *An. cruzii *sibling species. Very high *F*_*ST *_values (ranging from ~0.6 to 0.9) were found between Florianópolis and Itaparica using both the whole fragment as well as the NR blocks for all loci. Table 3 also shows the average number of nucleotide substitutions per site (*Dxy*), the number of net nucleotide substitutions per site between species (*Da*) and the distribution of the four mutually exclusive categories of segregating sites observed in each comparison: the number of exclusive polymorphisms for each species (*S*_1 _and *S*_2_), the number of shared polymorphisms (*S*_*s*_) and the number of fixed differences (*S*_*f*_). The *timeless *and the *cycle *loci were the only ones that shared polymorphisms between Florianópolis and Itaparica, albeit they were few (7 and 1 for the whole fragment, respectively). All loci presented a large number of fixed differences between the two species (Table 3).

**Table 3:** Genetic differentiation between Florianópolis and Itaparica.

Locus	*F*_*ST*_	P (*F*_*ST*_)	*Dxy*	*Da*	*S*_*s*_	*S*_*f*_	*S*_1_	*S*_2_
***timeless***	0.8150 (0.8144)	0.0000 (0.0000)	0.1154 (0.0877)	0.0941 (0.0714)	07 (01)	25 (15)	56 (17)	16 (12)
***Clock***	0.7088 (0.7500)	0.0000 (0.0000)	0.0593 (0.0579)	0.0420 (0.0434)	00 (00)	03 (04)	07 (06)	08 (06)
***cycle***	0.5806 (0.5852)	0.0000 (0.0000)	0.0371 (0.0441)	0.0215 (0.0258)	01 (00)	02 (02)	21 (13)	02 (03)
***Rp49***	0.8854 (0.8903)	0.0000 (0.0000)	0.0606 (0.0695)	0.0536 (0.0619)	00 (00)	12 (12)	10 (09)	09 (09)
***RpS2***	0.8502 (0.8598)	0.0000 (0.0000)	0.0843 (0.0836)	0.0717 (0.0718)	00 (00)	16 (16)	18 (18)	08 (07)
***RpS29***	0.8865 (0.9276)	0.0000 (0.0000)	0.0843 (0.0950)	0.0747 (0.0881)	00 (00)	11 (19)	14 (05)	19 (11)

*F*_*ST*_, pair-wise estimates of population differentiation. *P*-value, significance of *F*_*ST *_values (evaluated by 1,000 random permutations). *Dxy *and *Da*, average number of nucleotide substitutions per site and the number of net nucleotide substitutions per site between species, respectively [38]. *S*_1_, number of polymorphic sites exclusive to Florianópolis. *S*_2_, number of polymorphic sites exclusive to Itaparica. *S*_*s *_and *S*_*f*_, number of shared polymorphisms and number of fixed differences between the two species, respectively. Numbers in parentheses are related to the non-recombining block (NR) for each locus.

### Estimation of Demographic Population Parameters

The IM program was used to simultaneously estimate six demographic parameters (θ_1_, θ_2_, θ_*A*_, *t*, *m*_1_, *m*_2_) from the two *An. cruzii *sibling species through an "Isolation with Migration" model using multiple loci [11]. As mentioned above, only the NR blocks were used and some recombining sequences were removed before the IM analysis (Table 1).

**Figure 1:**
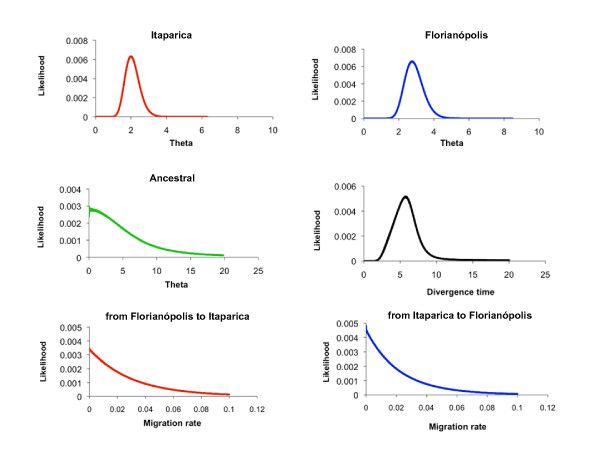
**Posterior probability distributions of demographic parameters**. Posterior probability distributions for each of the six demographic parameters estimated using IM: effective population size for an ancestral and two descendent populations (*theta*), divergence time between Florianópolis and Itaparica, and migration rates in both directions. Four IM simulations (a, b, c and d) using different seed numbers were plotted for each parameter estimate (see also Additional file 7). All curves are shown including the range of the priors.

Figure 1 shows the posterior probability distributions for each of the six demographic parameters estimated using IM and Additional file 7 summarizes the features from the marginal histograms for each of the parameters in all MCMC runs. Among four independent runs, the simulations between the two sibling species showed good convergence and consistency resulting in complete posterior distributions.

The estimates of *θ *suggest that the effective population size of the ancestral population is smaller than the current Florianópolis and Itaparica populations indicating that both may have had a history of growth since separation (Figure 1). The migration rates in both directions for all combined loci were also estimated by the IM software (*m*_1 _and *m*_2_). No indication of migration was found in either direction in the multiple simulations.

The divergence time parameter was estimated for all combined loci in four different IM runs. This parameter cannot be directly converted to years because the mutation rates in *Anopheles cruzii *species are unknown. Therefore, an estimate of the divergence time between *Anopheles cruzii *species was performed using the average of *Drosophila *synonymous and nonsynonymous substitution rates for several nuclear genes (0.0156 and 0.00191 per site per million year respectively) [12]. Using this approach and based on the average of HiSmth values, an estimate of the divergence time between Florianópolis and Itaparica would be approximately 2.4 *Mya *(range from 1.1 to 3.6 *Mya*, based on the average of HPD90Lo and HPD90Hi values).

Another manner of estimating the divergence time between these two *Anopheles *species is to use the same *Drosophila *synonymous substitution rate mentioned above and the average *Da *values from the six loci (Table 3). Based on these values, the divergence time between the populations from Florianópolis and Itaparica was estimated to be 1.91 ± 0.76 *Mya *and 1.93 ± 0.65 *Mya *for the whole sequence and NR blocks, respectively.

### Genealogy analysis

**Figure 2:**
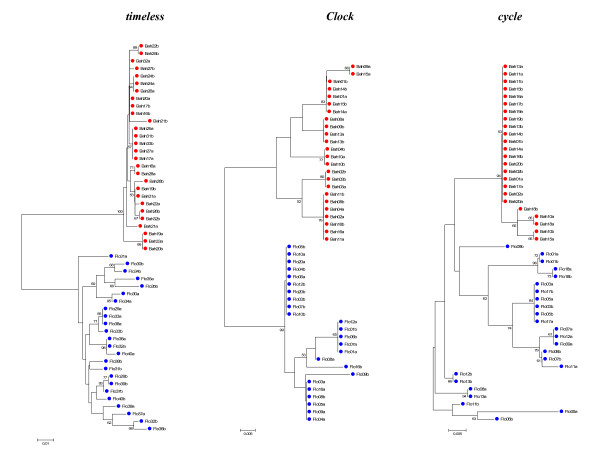
**Neighbor-joining trees of clock genes**. Neighbor-joining trees using the three clock gene nucleotide sequences of the two *An. cruzii *sibling species obtained with Jukes and Cantor distance for *Clock *gene, and Kimura 2-parameter distance for the others. Numbers on the nodes represent the percentage bootstrap values based on 1,000 replications. Flo: Florianópolis; Bah: Itaparica.

**Figure 3:**
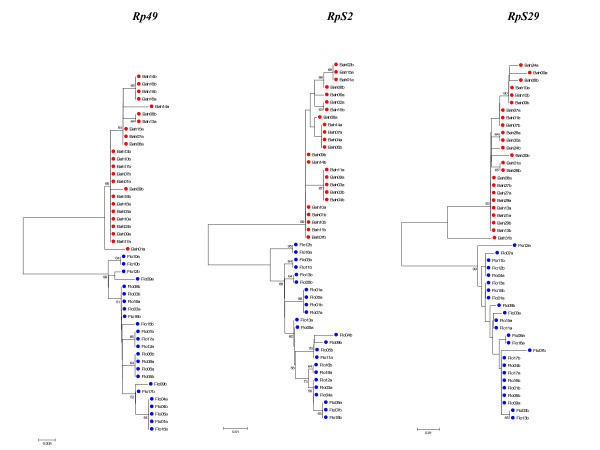
**Neighbor-joining trees of ribosomal protein genes**. Neighbor-joining trees using the three ribosomal protein gene nucleotide sequences of the two *An. cruzii *sibling species obtained with Kimura 2-parameter distance. Numbers on the nodes represent the percentage bootstrap values based on 1,000 replications. Flo: Florianópolis; Bah: Itaparica.

Gene trees of the sequences from all loci for both whole sequences and NR blocks were estimated using the Neighbor-Joining method (NJ) (Figures 2 and 3, and Additional files 8 and 9, respectively). The most suitable model selected using Modeltest 3.7 [13] was Kimura 2-parameter [14] for all loci except for the *Clock *gene where the Jukes and Cantor [15] model was chosen. All trees were performed with 1,000 bootstrap replicates. The resulting NJ trees clearly grouped the sequences from the two sibling species in different clusters with high bootstrap values in most cases.

## Discussion

The results presented here confirm the high level of differentiation between the Itaparica and Florianópolis sibling species of the *An. cruzii *complex [5,6].

Less differentiation might have been expected in the three genes that code for the highly conserved ribosomal proteins (*Rp49*, *RpS29 *and *RpS2*) than in loci possibly involved in the control of mating rhythms (*timeless*, *Clock *and *cycle*) [7,8]. The latter three genes are potentially important in maintaining temporal reproductive isolation between closely related species, and might be involved in the speciation process in some insects. In fact, Rona *et al*. [6] showed very high differentiation between Itaparica and the more southern Brazilian populations, including Florianópolis, using the *timeless *gene as a molecular marker.

However, very high *F*_*ST *_values were detected in all loci between these two sibling species and they were even higher for *Rp49*, *RpS29 *and *RpS2 *(0.8854, 0.8865 and 0.8502, respectively for the whole fragment) than for *timeless*, *Clock *and *cycle *(0.8150, 0.7088 and 0.5806, respectively for the whole fragment). Mazzoni *et al*. [16] found similar results in a multilocus analysis between two sand fly vectors of leishmaniasis.

No indication of migration was found in either direction in the multiple IM simulations, which was consistent with the very high differentiation values for all loci. Itaparica also presented lower levels of variability than those from Florianópolis, possibly indicating a smaller population size. This is confirmed by IM results, which also indicated a smaller effective population size for Itaparica. The estimated difference in population sizes seems coherent, since the southern *An. cruzii *sibling species found in Florianópolis is distributed throughout most of the southern and southeastern Brazilian Atlantic Forest (from Santa Catarina to Espírito Santo State) while the northeastern sibling species found in Itaparica seems to occur only in a more restricted region [6].

The multilocus results corroborate previous data [5,6] indicating that these populations represent two different species in the *An. cruzii *complex. This was also confirmed by NJ trees, which show that Florianópolis and Itaparica are clearly separated in two isolated groups, except perhaps in the case of *cycle *which suggests persistence of ancestral polymorphisms in Florianópolis. However, this gene fragment presents a very small number of variable sites in the Itaparica sample.

The estimated divergence time from 1.1 to 3.6 *Mya*, based on the IM results, corresponds to the end of the Pliocene and beginning of the Pleistocene [17]. Significant climate changes, including the onset of heavy Northern Hemisphere glaciation, around 2.75 *Mya*, occurred at the end of the Pliocene [18]. A very important consequence of this cooling was an extensive increase in aridification, which lead to fragmentation of forests, including the Brazilian Atlantic Forest [18,19]. Interestingly, Carnaval *et al*. [20] discussed the hypothesis of refugia for neotropical species occurring in the Atlantic Forest. Itaparica is located in an area proposed to be a large central refugium in the Brazilian Atlantic Forest and another refugium is proposed in the southern and southeastern Brazil. Climate changes have been proposed to explain the differentiation among many groups such as fruit flies [21], insect vectors [22] as well as many forest-obligate species [20,23-25]. Since *An. cruzii *is endemic to the Atlantic Forest, it seems likely that differentiation between its populations might have occurred due to forest fragmentation, which might have split a single ancestral species into two or more isolated groups.

## Conclusions

The results of the multilocus analysis corroborate previous data indicating that Florianópolis and Itaparica represent two different species of the *An. cruzii *complex and suggest that they have not exchanged migrants since their separation between 1.1 and 3.6 *Mya*.

## Methods

### Molecular analysis

The mosquitoes used in this study were females captured in Florianópolis, Santa Catarina State (SC) (27°31'S/48°30'W) and Itaparica Island (Jaguaripe), Bahia State (BA) (13°05'S/38°48'W). They were identified on the basis of their morphology according to Consoli and Lourenço-de-Oliveira [26]. For the molecular analysis, 12 individuals from Florianópolis and 12 to 14 from Itaparica were used for each gene.

The sequences of the *timeless *gene from Florianópolis and Itaparica were those previously published by our group [6] (Accession numbers: FJ408732 - FJ408865). The sequences of the other genes were obtained by PCR, cloning and sequencing as described below.

The primers listed in Table 4 were used with *An. cruzii *genomic DNA extracted according to Jowett [27] in PCR reactions carried out in an Eppendorf Mastercycler^® ^thermocycler using the proofreading Pfu DNA polymerase (Biotools). PCR products were purified and cloned using either Zero Blunt TOPO PCR cloning kit (Invitrogen) or pMOS Blue vector blunt-ended cloning kit (GE Healthcare). Sequencing of positive clones was carried out in an ABI Prism 3730 DNA sequencer at the Oswaldo Cruz Institute using the ABI Prism Big Dye Terminator Cycle Sequencing Ready Reaction kit (Applied Biosystems). The identity of the cloned fragments was determined by BlastX analysis using GenBank [28].

**Table 4:** Sequence of primers used to amplify the gene fragments

Locus	Primers name	Sequence of primers
***Clock***	5'CLKdeg3	5'-SNGGNTAYGAYTAYTAYCA-3'
	3'CLKdeg10	5'-TCNGTYTGNARCCADATCCA-3'
	5'cruziiclock	5'-TTGACGATCTGGAAAAGGTG-3'
	3'cruziiclock	5'-CTTGGTCAGGAAGCGATAGT-3'

***cycle***	5'CYCdeg1	5'-ARMGNMGNMGNGAYAARATGAA-3'
	3'CYCdeg1	5'-ACYTTNCCDATRTCYTTNGGRTG-3'
	5'cruziicycle	5'-CACCTACATCACCGAACTG-3'
	3'cruziicycle	5'-GACTCGGAAACGTACAGGATA-3'

***Rp49***	5'aquaRP1	5'-GTGAAGAAGCGGACGAAGAAGTT-3'
	3'aeaquaRP1b	5'-TCATCAGCACCTCCAGCTC-3'

***RpS2***	5'cruziiRP_S2	5'-GGCTACTGGGGTAACAAGA-3'
	3'cruziiRP_S2	5'-CAGRACGGAACCGCACTT-3'

***RpS29***	5'cruziiRP_S29b	5'-TCGCATCCSCGTAAATA-3'
	3'cruziiRP_S29	5'-TTCCKGAAGCCAATATCCT-3'

Degenerate and specific primers used to amplify the different gene fragments in the two *An. cruzii *sibling species.

The sequences of primer pairs 5'CYCdeg1 + 3'CYCdeg1 and 5'aquaRP1 + 3'aeaquaRP1b are from [39] and [40], respectively. Degenerated primers were used in preliminary amplifications to isolate initial fragments of the *cycle *and *Clock *genes. Sequence of these fragments allowed the design of the specific primers used in the population genetics analysis.

At least eight clones were sequenced for each mosquito. Sequences were edited and in most cases consensus sequences representing the two alleles were generated. In a number of individuals only one haplotype was observed among the eight sequences and in these cases mosquitoes were classified as homozygotes. The probability of incorrectly classifying a heterozygote as a homozygote individual with this procedure is less than 1%. The sequences from homozygote mosquitoes were duplicated prior to analysis. However, when carried out without duplicating homozygote sequences, the analysis rendered similar results. Sequences were submitted to GenBank (Accession numbers: GU016330-GU016569).

### DNA sequence analysis

The sequences were aligned using ClustalX software [29] and population genetics analysis was carried out using DNASP4.0 [30] and P_RO_S_EQ _v 2.91 [31] softwares.

The Modeltest version 3.7 [13] was used with a model block implemented in PAUP 4.0d105 [32] to find the most suitable model for each gene evolution. Models selected by the Bayesian Information Criterion (BIC) were favored and used in the phylogenetic analysis, carried out using MEGA 4.0 [33].

The IM program is an implementation of the Isolation with Migration model and is based on the MCMC (Markov Chain Monte Carlo) simulations of genealogies [11,34]. It simultaneously estimates six demographic parameters from multilocus data: effective population size for an ancestral and two descendent populations (*θ*_A_, *θ*_1_, and *θ*_2_, respectively), divergence time (*t*) and migration parameters in both directions (*m*_1 _and *m*_2_). Initial IM runs were performed in order to establish appropriate upper limits for the priors of each demographic parameter. These preliminary simulations generated marginal distributions that facilitated the choice of parameter values used in the final IM analyses. The convergence was assessed through multiple long runs (four independent MCMC runs with different seed numbers were carried out with at least 30,000,000 recorded steps after a burn-in of 100,000 steps) and by monitoring the ESS values, the update acceptance rates and the trend lines.

The Infinite Sites model [35] was chosen as the mutation model in the IM simulations because the two species are closely related and all genes are nuclear.

The optimal recombination-filtered block was extracted from each gene alignment using the IM_GC _program, which also removes haplotypes that represent likely recombinant sequences [36,37].

## Competing interests

The authors declare that they have no competing interests.

## Authors' contributions

LDPR participated in data generation and analysis, and drafted the manuscript. She also helped capture mosquitoes in Florianópolis. CJCP carried out the capture and morphological identification of mosquitoes. CJM helped in the IM analysis. AAP is the principal investigator, participated in its design and coordination, and helped to write the manuscript. All authors read and approved the final manuscript.

## Supplementary Material

Additional file 1 **Alignment of the *timeless *sequences from Florianópolis and Itaparica**. Alignment of the DNA sequences from the *timeless *gene fragment from Florianópolis and Itaparica. The translated amino acid sequence is shown above the alignment and the intron is highlighted in grey. Dots represent identity and dashed represent gaps. The asterisks in the bottom line represent identity of all sequences. Flo: individuals from Florianópolis and Bah: individuals from Itaparica. Click here for file

Additional file 2 **Alignment of the Clock sequences from Florianópolis and Itaparica**. Alignment of the DNA sequences from the *Clock *gene fragment from Florianópolis and Itaparica. The translated amino acid sequence is shown above the alignment and the intron is highlighted in grey. Dots represent identity and dashed represent gaps. The asterisks in the bottom line represent identity of all sequences. Flo: individuals from Florianópolis and Bah: individuals from Itaparica. Click here for file

Additional file 3 **Alignment of the *cycle *sequences from Florianópolis and Itaparica**. Alignment of the DNA sequences from the *cycle *gene fragment from Florianópolis and Itaparica. The translated amino acid sequence is shown above the alignment. Dots represent identity and dashed represent gaps. The asterisks in the bottom line represent identity of all sequences. Flo: individuals from Florianópolis and Bah: individuals from Itaparica. Click here for file

Additional file 4 **Alignment of the *Rp49 *sequences from Florianópolis and Itaparica**. Alignment of the DNA sequences from the *Rp49 *gene fragment from Florianópolis and Itaparica. The translated amino acid sequence is shown above the alignment and the intron is highlighted in grey. Dots represent identity and dashed represent gaps. The asterisks in the bottom line represent identity of all sequences. Flo: individuals from Florianópolis and Bah: individuals from Itaparica. Click here for file

Additional file 5 **Alignment of the *RpS2 *sequences from Florianópolis and Itaparica**. Alignment of the DNA sequences from the *RpS2 *gene fragment from Florianópolis and Itaparica. The translated amino acid sequence is shown above the alignment and the intron is highlighted in grey. Dots represent identity and dashed represent gaps. The asterisks in the bottom line represent identity of all sequences. Flo: individuals from Florianópolis and Bah: individuals from Itaparica. Click here for file

Additional file 6 **Alignment of the *RpS29 *sequences from Florianópolis and Itaparica**. Alignment of the DNA sequences from the *RpS29 *gene fragment from Florianópolis and Itaparica. The translated amino acid sequence is shown above the alignment and the intron is highlighted in grey. Dots represent identity and dashed represent gaps. The asterisks in the bottom line represent identity of all sequences. Flo: individuals from Florianópolis and Bah: individuals from Itaparica. Click here for file

Additional file 7 **Summarized features of the marginal histograms for each parameter**. Values of the six parameters that span the prior distribution are presented for each of the four runs with different seed numbers (a, b, c and d). Population size parameter for Itaparica, Florianópolis and ancestral populations (θ_1_, θ_2_, θ_*A*_); Time of population splitting parameter (*t*); Migration rate estimate from Florianópolis to Itaparica population (*m*_1_) and from Itaparica to Florianópolis population (*m*_2_); Minbin and Maxbin, the midpoint values of the lowest and the highest bin, respectively; HiPt, the value of the bin with the highest count; HiSmth, the value of the bin with the highest count, after the counts have been smoothed by taking a running average of 9 points centered on each bin; 95Lo and 95Hi, the estimated points to which 2.5% of the total area lies to the left and to the right, respectively; HPD90Lo and HPD90Hi, the lower and upper bounds of the estimated 90% highest posterior density (HPD) interval, respectively. Click here for file

Additional file 8 **Neighbor-joining trees of NR blocks of clock genes**. The trees were estimated using the neighbor-joining method with 1,000 bootstrap replicates. The Jukes and Cantor distance was used for the *Clock *gene and Kimura 2-parameter distance for the others. Click here for file

Additional file 9 **Neighbor-joining trees of NR blocks of ribosomal protein genes**. The trees were estimated using the neighbor-joining method with 1,000 bootstrap replicates and Kimura 2-parameter distance. Click here for file
