# Supplementary material for: Estimation of divergence time between two sibling species of the Anopheles (Kerteszia) cruzii complex using a multilocus approach
Source: BMC Evol Biol. 2010 Mar 31;10:91. doi: 10.1186/1471-2148-10-91 (PMC3087556; doi:10.1186/1471-2148-10-91)
Supplement: Additional file 1 — Alignment of the timeless sequences from Florianópolis and Itaparica. Alignment of the DNA sequences from the timeless gene fragment from Florianópolis and Itaparica. The translated amino acid sequence is shown above the alignment and the intron is highlighted in grey. Dots represent identity and dashed represent gaps. The asterisks in the bottom line represent identity of all sequences. Flo: individuals from Florianópolis and Bah: individuals from Itaparica. [file 1471-2148-10-91-S1.DOC]

00000000000000000000000000000000000000000000000000000000000000000000000000000000000000000000000000011111111111111111111111111111111111111111111111111111111111111111111111111111111111111111111111111112222222222222222222222222222222222222222222222222222222222222222222222222222222222222222222222222222333333333333333333333333333333333333333333333333333333333333333333333333333333333333333333333333333344444444444444

00000000011111111112222222222333333333344444444445555555555566666666677777777778888888888999999999900000000001111111111222222222233333333334444444444555555555566666666667777777777888888888899999999990000000000111111111122222222223333333333444444444455555555556666666666777777777788888888889999999999000000000011111111112222222222333333333344444444445555555555666666666677777777778888888888999999999900000000001111

12345678901234567890123456789012345678901234567890123456789012345678901234567890123456789012345678901234567890123456789012345678901234567890123456789012345678901234567890123456789012345678901234567890123456789012345678901234567890123456789012345678901234567890123456789012345678901234567890123456789012345678901234567890123456789012345678901234567890123456789012345678901234567890123456789012345678901234567890123

N P A P A Q E K K E L R R K K L V K R G K S N I I N M K G L M H H V P T D D D I S H I L K E F T V D F L L K G Y G Y L V Q E L H S Q L L S D L

Bah27b AAATCCCACGTCGGCCCTGGAGAAGAAGGAGCTGCGGCGCAAGAAGCTGGTGAAGCGGGGCAAGAGCAACATGTCAGTAGGAACCGGAACCGGC--CGCGTTCGCAGTTCGCTGGTAGTAACTGGTAGTTTCGATTTCCAACCCTTTCTCCAGCATCAACATGAAGGGCCTGATGCACCACGTCCCGTCGGACGACGACATATCGCATATCCTGAAGGAGTTCACGGTCGACTTTCTGCTCAAAGGCTACGGCTATCTGGTGCAGGAGTTGCACACCCAGCTGCTGTCGGATTTGGTGAGTCCTG------TGGT-------CCTGTGGTTTGTGGAGAAATGCCCCCTGTGCCGTTGTTTAATCATTCCCCCTTCGAATCCCCCTTTCCGGCGTTC--ACTTTAT--ACAGC

Bah16a C.............................................................................................--...................................T.............................................................................................................................................................................------....-------..........................................T................................--.......--.....

Bah16b C.............................................................................................--.................................................................................................................................................................................................................------....-------...........................................................................--.......--.....

Bah17a C.............................................................................................--.................................................................................................................................................................................................................------....-------..................................................T........................--.....T.--.....

Bah17b C.............................................................................................--.................................................................................................................................................................................................................------....-------...........................................................................--.......--.....

Bah19a C........A.....G.A............................................................................--.................................................................................................................................................................................................................------....-------..........A.............................................................C..--.....T.--.....

Bah19b C.............................................................................................--...................................T..................T..........................................................................................................................................................------....-------...........................................................................--.......--.....

Bah20a C.............................................................................................--.................................................................................................................................................................................................................------....-------...........................................................................--.......--.....

Bah20b C........A.....G.A............................................................................--.................................................................................................................................................................................................................------....-------..........A.............................................................C..--.....T.--.....

Bah21a C.............................................................................................--..................A......................................................................................................................................T.......................................................------....-------..........T................................................................--.......--.....

Bah21b C.....T.......................................................................................--........................................................................................................................A........................................................................................------....-------..................G...T...............................A....................--.......--.....

Bah22a C.............................................................................................--...................................T..................T..................T.......................................................................................................................................------....-------...........................................................................--.......--.....

Bah22b C.............................................................................................--................................................................................................T................................................................................................................------....-------.........................T.................................................--.......--.....

Bah24a C.............................................................................................--...............................................................................................................................................................C.................................................------....-------...........................................................................--.......--.....

Bah24b C.............................................................................................--...............................................................................................................................................................C.................................................------....-------...........................................................................--.......--.....

Bah25a C.............................................................................................--.................................................................................................................................................................................................................------....-------..................................................T........................--.....T.--.....

Bah25b C.............................................................................................--................................................................................................T................................................................................................................------....-------.........................T.................................................--.......--.....

Bah26a C.............................................................................................--...............................................................................................................................................................C.................................................------....-------...........................................................................--.......--.....

Bah26b C.............................................................................................--..T................................T..................T..........................................................................................................................................................------....-------...........................................................................--.......--.....

Bah27a C.............................................................................................--.................................................................................................................................................................................................................------....-------..................................................T........................--.....T.--.....

Bah28a C.............................................................................................--...................................T.............................................................................................................................................................................------....-------..........................................T................................--.......--.....

Bah28b C.............................................................................................--...................................T..................T..........................................................................................................................................................------....-------..........T................................................T...............--.......--.....

Bah31a C.............................................................................................--...................................T..................T..........................................................................................................................................................------....-------...........................................................................--.......--.....

Bah31b C.............................................................................................--.................................................................................................................................................................................................................------....-------..................................................T........................--.....T.--.....

Bah32a C.............................................................................................--.................................................................................................................................................................................................................------....-------...........................................................................--.......--.....

Bah32b C.............................................................................................--..T................................T..................T..........................................................................................................................................................------....-------...........................................................................--.......--.....

Bah33a C........A.....G.A............................................................................--.................................................................................................................................................................................................................------....-------..........A.............................................................C..--.....T.--.....

Bah33b C.............................................................................................--.................................................................................................................................................................................................................------....-------..................................................T........................--.....T.--.....

Flo29a C......G..C....G.AA...........A..........................................................AG...GCTC.AG....G.C...T.CACC.C.....--..A..T.T.........T...--..........T...........................A.......T................................T.......................................C.....T.....T........................GTCCGA....TCACTTT....C.............-G.TA......T...G.A............G--..........T.......A.....CG.......CC.....

Flo29b C......G..C....G.A............A..........................................................AG...GCTC.AA....G.C...T.CACC.C....A--..A..T.T.........T...--..........T...........................A.......T................................T.......................................C.....T.....T........................GTCCGA....TCACTTT....C......C......-G.TA..........G.T............G--.......A..T.......A.....CG.......CC.....

Flo30a C......G..C...TT.A............A.....A....................................................AG...GCTC.AG..C.G.C...T.CACC.......--..A..T.T.........T...T-T.........T.......................T...A.......T................................T.......................................C.....T.....T........................GTCCGA....TCACTTT.-..C.............-..TA....TC....G.TC...........G--..........T.............--.......CC.....

Flo30b C......G..C....T.A............A.................................................T........AG...GCTC.AG..C.G.C...T.CACC.......--..A..T.T.........T...T-..........T.......................T...A.......T................................T.......................................C.....T.....T........................GTCCGA....TCACTTT....C.............-..TA.....C....G.TC.....T.....G--.........TT.......A.....CG...C...CC.....

Flo31a C......G..C....G.A............A..........................................................AG...GCTC.AG....G.C...T.CACC.C.....--..A..T.T.........T...--..........T...........................A.......T........................................................................C.....T.....T........................GTCCGA....TCACTTT....CA.......A....-..TA.......A..G.T.......G....G--..........T.C...T.......--.......CC.....

Flo31b C......G..C....G.A............A.......................A..................................AG...GCTC.AG....G.C...T.CACC.C.....--..A..T.T.........T...--..........T...........................A.......T........................................................................C.....T.....T........................GTCCGA....TCACTTTT...C.............-..TA..........G.T......A.....G--........T.T.......A.....CA.......CC.....

Flo32a C......G..C....G.A............A..........................................................AG...GC.C.AG..C.G.C...T.CACC.C.....--..A..T.T.........T...--..........T...........................A.......T................................T.......................................C.....T.....T........................GTCCGA....TCACTTT....C.............-G.TA.......A..G.T............G--...C......T.....G.A.....CG.......CG.....

Flo32b C......G..C....G.A.........A..A..........................................................AG...GCTC.AG....G.C...T.CACC.C.....--..A..T.T.........T...--..........T........T.................AA.......T................................T.......................................C.....T.....T.....................G..GTCCGA....TCACTTT....C......C......-GATA..........G.T.C....A.....G--..TC......T.......A.....CG.......CC.....

Flo33a C......G..C....G.AA...........A..........................................................AG...GCTC.AG....G.C...T.CACC.C.....--..A..T.T.........T...--..........T...........................A.......T................................T.......................................C.....T.....T........................GTCCGA....TCACTTT....C.............-G.TA......T...G.A............G--..........T.......A.....CG.......CC.....

Flo33b C......G..C....G.AA...........A..........................................................AG...GC.C.AG..C.G.C...T.CACC.C.....--..A..T.T.........T...--..........T...........................A.......T................................T.......................................C.....T.....T........................GTCCGA....TCACTTT....C.............-G.TA......T...G.A............G--..........T.......A.....CG.......CC.....

Flo34a C......G..C......A............A.....A....................................................AG...GCTC.AG..C.G.C...T.CACC.......--..A..T.T.........T...T-T.........T.......................T...A.......T................................T.......................................C.....T.....T........................GTCCGA....TCACTTT.-..C.............-..TA....TC....G.TC...........G--..........T.............--.......CC.....

Flo34b C......G..C......AA...........A..........................................................AG...GCTC.AG..C.G.C...T.CACC.C.....--..A..T.T.........T...--..........T.......................T...A.......T................................T.......................................C.....T.....T........................GTCCGA....TCACTTT....C.............-..TA.....C....G.TC.....T.....G--.........TT.......A.....CG...C...CC.....

Flo35a C......G..C......AA...........A.....A....................................................AG...GCTC.AG..C.G.C...T.CACC.C.....--..A..T.T.........T...--..........T.......................T...A.......T................................T.......................................C.....T.....T........................GTCCGA....TCACTTT...CC..........A..-G.TA...G......G.TC...........G--...C......T.......A.....CG.T.....CC.....

Flo35b C......G..C......AA...........A.................................................A........AG...GCTCTAG..C.G.C...T.CACC.C.....--..A..T.T.........T...--..........T........T..............T...A.......T................................T.............................................T.....T........................GTCCGA....TCACTTT...CC..........A..-G.TA..........G.TC...........G--.........TT.......A.....CG...C...CC.....

Flo36a C......G..C....G.A............A..........................................................AG...GC.C.AG..C.G.C...T.CACC.C.....--..A..T.T.........T...--..........T...........................A.......T................................T.......................................C.....T.....T........................GTCCGA....TCACTTT....C.............-G.TA.......A..G.T............G--...C......T.....G.A.....CG.......CG.....

Flo36b C......G.AC....G.AA...........A..........................................................AG...GCTC.AG....G.C...T.CACC.C.....--..A..T.T.........T...--..........T........T.................AA.......T................................T....................T..................C.....T.....T.....................G..GTCCGA....TCACTTT....C......C......-GATA..........G.T.C....A.....G--..TC......T.......A.....CG.......CC.....

Flo37a C.........C....G.A............A..........................................................AG...GCTC.AG..C.G.C...T.CACC.C.....--..A..T.T.........T...--..........T...........................A.......T................................T..T....................................C.....T.....T........................GTCCGA....TCACTTT....C......C......-G.TA..........G.T.C....A.....G--..TC..C...T.......A.....CG.......CC.....

Flo37b C......G..C....G.A............A..........................................................AG...GCTC.AA....G.C...T.CACC.C....A--..A..T.T.........T...--..........T...........................A.......T................................T.......................................C.....T.....T........................GTCCGA....TCACTTT....C......C......-G.TA..........G.T............G--..........T.......A.....CG.......CC.....

Flo38a C......G..C....G.AA...........A..........................................................AG...GCTC.AG....G.C...T.CACC.C.....--..A..T.T.........T...--..........T...........................A.......T................................T.......................................C.....T.....T........................GTCCGA....TCACTTT....C.............-G.TA......T...G.A............G--..........T.......A.....CG.......CC.....

Flo38b C......G..C....G.A.........A..A..........................................................AG...GCTC.AG....G.C...T.CACC.C.....--..A..T.T.........T...--..........T...........................A.......T................................T.......................................C.....T.....T........................GTCCGA.A..TCACTTTT...C.............-..TA..........G.T............G--..........T.......A.....CG.......CC.....

Flo39a C......G..C....G.A............A...........................................................G...GCTC.AG..C.G.C...T.CACC.C.....--..A..T.T.........T...--..........T........T..................A.......T........................................................................C.....T.....T........................GTCCGA....TCACTTTT...C.............-..TA..........G.T.C....A.....G--..TC......T.......A.....CG.......CC.....

Flo39b C......G..C....G.A............A..........................................................AG...GCTC.AA....G.C...T.CACC.C....A--..A..T.T.........T...--..........T...........................A.......T................................T.......................................C.....T.....T........................GTCCGA....TCACTTT....C......C......-G.TA..........G.T............G--.......A..T.......A.....CG.......CC.....

Flo40a C......G..C....G.A............A..........................................................AG...GC.C.AG..C.G.C...T.CACC.C.....--..A..T.T.........T...--..........T...........................A.......T................................T.........................CC............C.....T.....T........................GTCCGA....TCACTTT....C.............-G.TA.......A..G.T............G--...C......T.....G.A.....CG.......CG.....

Flo40b C......G..C....G.A.........A..A..........................................................AG...GCTC.AG....G.C...T.CACC.C.....--..A..T.T.........T...--..........T...........................A.......T................................T.......................................C.....T.....T........................GTCCGA....TCACTTT....C......C......-G.TA..........G.T......A.....G--..........T.......A.....CG.......CC.....

***** * *** * ******** ** ***** ***************** ************************* ******** *** ** * * *** * * **** ** ** * ********* *** ******** ******** ************* ** **** ** ******************** *********** ** ***************** **** ************ ***** ***** ********************* ** * ** * **** * * ** ** ** * **** **** ** ** * *** * ** ** * * * * *****
