# Supplementary material for: Estimation of divergence time between two sibling species of the Anopheles (Kerteszia) cruzii complex using a multilocus approach
Source: BMC Evol Biol. 2010 Mar 31;10:91. doi: 10.1186/1471-2148-10-91 (PMC3087556; doi:10.1186/1471-2148-10-91)
Supplement: Additional file 2 — Alignment of the Clock sequences from Florianópolis and Itaparica. Alignment of the DNA sequences from the Clock gene fragment from Florianópolis and Itaparica. The translated amino acid sequence is shown above the alignment and the intron is highlighted in grey. Dots represent identity and dashed represent gaps. The asterisks in the bottom line represent identity of all sequences. Flo: individuals from Florianópolis and Bah: individuals from Itaparica. [file 1471-2148-10-91-S2.DOC]

000000000000000000000000000000000000000000000000000000000000000000000000000000000000000000000000000111111111111111111111111111111111111111111111111111111111111

000000000111111111122222222223333333333444444444455555555555666666666777777777788888888889999999999000000000011111111112222222222333333333344444444445555555555

123456789012345678901234567890123456789012345678901234567890123456789012345678901234567890123456789012345678901234567890123456789012345678901234567890123456789

V A C H E A L M Q K G E G T S C

Bah16a GTGGCTTGTCATGAAGCATGTAAGCGCTACAATACCTCTACTCTTGTAATCTCCCGCCCGGGCTGCTCTCAATGATTTAAAT-CCGTTTCTTTTCTTCCGTTGG---------------------GCAGTGATGCAAAAGGGCGAGGGAACTTCGTGCT

Bah16b ..................................................................................-.....................---------------------..................................

Bah01a ........C......................................G....T.............................-.....C...............---------------------..................................

Bah01b ........C......................................G....T.............................-.....C...............---------------------..................................

Bah02a ..................................................................................-.....................---------------------..................................

Bah02b ............................................................A.....................-.....................---------------------.............................C....

Bah03a ............................................................A.....................-.....................---------------------.............................C....

Bah03b ............................................................A.....................-.....................---------------------.............................C....

Bah04a ..................................................................................-.....................---------------------..................................

Bah04b ...............................................G....T.............................-.....................---------------------.A...........................C....

Bah08a ...............................................G....T.............................-.....C...............---------------------.............................C....

Bah08b ..................................................................................-.....................---------------------..................................

Bah09a ........C......................................G....T.............................-.....C...............---------------------..........................C.......

Bah09b ...............................................G....T.............................-.....C...............---------------------.............................C....

Bah10a ...............................................G....T.............................-.....................---------------------.A...........................C....

Bah10b ...............................................G....T.............................-.....................---------------------.A...........................C....

Bah11a ..................................................................................-.....................---------------------..................................

Bah11b ..................................................................................-.....................---------------------..................................

Bah13a ...............................................G....T.............................-.....C...............---------------------.............................C....

Bah13b ...............................................G....T.............................-.....C...............---------------------.............................C....

Bah14a ........C......................................G....T.............................-.....C...............---------------------..................................

Bah14b ........C......................................G....T.............................-.....C...............---------------------..................................

Bah15a ........C......................................G....T.............................-.....C...............---------------------..........................C.......

Bah15b ........C......................................G....T.............................-.....C...............---------------------..................................

Flo01a ...................................T...........G....T..C..T.A.G-..G......A........-.....................TACGCTTTCTCTCCGCGTGCT..................................

Flo01b ...................................T...........G....T..C..T.A.G-..G......A........-.....................TACGCTTTCTCTCCGCGTGCT..................................

Flo03a .................................-.T...........G....T..C..T.A.G-..................-...G.................TGCGGTTTCTCTCCGCGTGCT..................................

Flo03b ...................................T...........G....T..C..T.A.G-..................-.....................TGCGGTTTCTCTCCGCCTGCT..................................

Flo04a ...................................T...........G....T..C..T.A.G-..................-...G.................TGCGGTTTCTCTCCGCGTGCT..................................

Flo04b ...................................T...........G....T..C..T.A.G-..................-.....................TGCGGTTTCTCTCCGCCTGCT..................................

Flo05a ...................................T...........G....T..C..T.A.G-..................-...G.................TGCGGTTTCTCTCCGCGTGCT..................................

Flo05b ...................................T...........G....T..C..T.A.G-..................-.....................TGCGGTTTCTCTCCGCCTGCT..................................

Flo06a ...................................T...........G....T..C..T.A.G-..................-.....................TGCGGTTTCTCTCCGCCTGCT..................................

Flo06b ...................................T...........G....T..C..T.A.G-..G......A........-.....................TACGCTTTCTCTCCGCGTGCT..................................

Flo07a ...................................T...........G....T..C..T.A.G-..G......A........-.....................TACGCTTTCTCTCCGCGTGCT..................................

Flo07b ...................................T...........G....T..C..T.A.G-..................-.....................TGCGGTTTCTCTCCGCCTGCT..................................

Flo08a .................................-.T...........G....T..C..T.A.G-..G...............-.....................TGCGGTTTCTCTCCGCGTGCT..................................

Flo08b ...................................T...........G....T..C..T.A.G-..................-...G.................TGCGGTTTCTCTCCGCGTGCT..................................

Flo09a .................................-.T...........G....T..C..T.A.G-..................-...G.................TGCGGTTTCTCTCCGCGTGCT..................................

Flo09b ...................................T...........G....T..T..T.A.G-.....T............-...G.................TGCGGTTTCTTTCCGCGTGCT..................................

Flo10a ...................................T...........G....T..C..T.A.G-..................-.....................TGCGGTTTCTCTCCGCCTGCT..................................

Flo10b ...................................T...........G....T..C..T.A.G-..................-.....................TGCGGTTTCTCTCCGCCTGCT..................................

Flo12a .................................-.T...........G....T..C..T.A.G-..G......A........-.....................TACGCTTTCTCTCCGCGTGCT..................................

Flo12b ...................................T...........G....T..C..T.A.G-..................-.....................TGCGGTTTCTCTCCGCCTGCT..................................

Flo16a .................................-.T...........G....T..C..T.A.G-..................-...G.................TGCGGTTTCTCTCCGCGTGCT..................................

Flo16b ..................................TT...........G....T..C..T.A.G-..G...............T...G.................TGCGGTTTCTCTCCGCGTGCT..................................

Flo20a ...................................T...........G....T..C..T.A.G-..................-.....................TGCGGTTTCTCTCCGCCTGCT..................................

Flo20b ...................................T...........G....T..C..T.A.G-..................-.....................TGCGGTTTCTCTCCGCCTGCT..................................

******** ************************ *********** **** ** ** * * ** ** *** ******** *** * *************** * ************************ ** ****
