# Supplementary material for: Estimation of divergence time between two sibling species of the Anopheles (Kerteszia) cruzii complex using a multilocus approach
Source: BMC Evol Biol. 2010 Mar 31;10:91. doi: 10.1186/1471-2148-10-91 (PMC3087556; doi:10.1186/1471-2148-10-91)
Supplement: Additional file 3 — Alignment of the cycle sequences from Florianópolis and Itaparica. Alignment of the DNA sequences from the cycle gene fragment from Florianópolis and Itaparica. The translated amino acid sequence is shown above the alignment. Dots represent identity and dashed represent gaps. The asterisks in the bottom line represent identity of all sequences. Flo: individuals from Florianópolis and Bah: individuals from Itaparica. [file 1471-2148-10-91-S3.DOC]

00000000000000000000000000000000000000000000000000000000000000000000000000000000000000000000000000011111111111111111111111111111111111111111111111111111111111111111111111111111111111111111111111111112222222222222222222

00000000011111111112222222222333333333344444444445555555555566666666677777777778888888888999999999900000000001111111111222222222233333333334444444444555555555566666666667777777777888888888899999999990000000000111111111

12345678901234567890123456789012345678901234567890123456789012345678901234567890123456789012345678901234567890123456789012345678901234567890123456789012345678901234567890123456789012345678901234567890123456789012345678

S A M I P M C H A M S R K L D K L T V L R M A V Q H L K T I R G A V H S Y T E G H Y K P A F L S D Q E L K M L I L Q A A E G F L F V V G C D R G

Bah10a TCCGCGATGATACCGATGTGCCACGCGATGTCCCGCAAGCTGGACAAGCTGACGGTGCTGCGGATGGCCGTGCAGCATCTGAAGACGATCCGCGGTGCGGTCCACTCGTACACGGAGGGCCACTACAAGCCGGCCTTTCTGTCGGACCAGGAGCTCAAGATGCTGATCCTGCAGGCGGCCGAAGGCTTCCTGTTCGTGGTCGGGTGCGATCGGGGCCG

Bah10b .............................................................................C.........................................T..................................................................................................

Bah01a .............................................................................C.................C..........................................................................................................................

Bah01b .............................................................................C.................C..........................................................................................................................

Bah02a .............................................................................C.................C..........................................................................................................................

Bah02b .............................................................................C.................C..........................................................................................................................

Bah11a .............................................................................C.................C..........................................................................................................................

Bah11b .............................................................................C.................C..........................................................................................................................

Bah13a .............................................................................C.................C..........................................................................................................................

Bah13b .............................................................................C.................C..........................................................................................................................

Bah14a .............................................................................C.................C..........................................................................................................................

Bah14b .............................................................................C.................C..........................................................................................................................

Bah15a .............................................................................C.........................................T..................................................................................................

Bah15b .............................................................................C.................C..........................................................................................................................

Bah16a .............................................................................C.................C..........................................................................................................................

Bah16b .............................................................................C.................C..........................................................................................................................

Bah17a .............................................................................C.................C..........................................................................................................................

Bah17b .............................................................................C.................C..........................................................................................................................

Bah18a ..........................................................................................................................................................................................................................

Bah18b .............................................................................C............................................................................................................................................

Bah19a .............................................................................C.................C..........................................................................................................................

Bah19b .............................................................................C.................C..........................................................................................................................

Bah20a .............................................................................C.................C..........................................................................................................................

Bah20b .............................................................................C.................C..........................................................................................................................

Flo01a ...................................A..........................A..............C.................C..A..........................T.....A...........C.............................A.........................................T..

Flo01b ...................................A..........................A..............C.................C..A..........................T.....A...........C.............................A.........................................T..

Flo03a ...................................A.........................................C.................C..A..T.............................A...........C............................................T..............T...........T..

Flo03b ...................................A.........................................C.................C..A..T.............................A...........C............................................T..............T...........T..

Flo05a ...................................A.........................................C.................C..A..T.............................A...........C............................................T..............T...........T..

Flo05b ...................................A.........................................C.................C..A..T.............................A...........C............................................T..............T...........T..

Flo06a ..........................A........A................................G........C........A.....T..C..A..T..............A...........A..........................G................................T..........................T..

Flo06b ..........................A........A....................A....................C..............T..C..A..T.....................................................G...........................................................T..

Flo07a ...................................A....................A....................C...........T.....C..A................................A...........C............................................T..............T...........T..

Flo07b ...................................A....................A....................C...........T.....C..A................................A...........C............................................T..........................T..

Flo08a ...................................A................................T........C.................C..A...........T............................................G...........................................................T..

Flo08b ...................................A....................A....................C...........T.....C..A................................A...........C............................................T..........................T..

Flo09a ...................................A....................A....................C...........T.....C..A................................A...........C............................................T..............T...........T..

Flo09b ...................................A................................G........C..............T..C..A..T.........................................C..........................................................................

Flo11a ...................................A....................A...A................C...........T.....C..A................................A...........C............................................T..........................T..

Flo11b ...................................A.........................................C.................C..A..T.....................................................G................................T..........................T..

Flo12a ...................................A....................A....................C...........T.....C..A................................A...........C............................................T..............T...........T..

Flo12b ...................................A.........................................C.................C..A..T.....................................................G..............................................................

Flo13a ...................................A................................T........C.................C..A...........T............................................G...........................................................T..

Flo13b ...................................A.........................................C.................C..A..T.....................................................G..............................................................

Flo17a ...................................A.........................................C.................C..A..T.............................A...........C............................................T..............T...........T..

Flo17b ...................................A.........................................C.................C..A..T.............................A...........C............................................T..............T...........T..

Flo18a ...................................A..........................A..............C....................A..........................T.....A...........C.............................A.........................................T..

Flo18b ...................................A..........................A..............C....................A..........................T.....A...........C.............................A.........................................T..

************************** ******** ******************** *** * ***** ******** ******** ** ** ** ** ** ******** ***** ** ***** ** ** *********** *********** ***************** ************** ************** *********** **
