# Supplementary material for: Estimation of divergence time between two sibling species of the Anopheles (Kerteszia) cruzii complex using a multilocus approach
Source: BMC Evol Biol. 2010 Mar 31;10:91. doi: 10.1186/1471-2148-10-91 (PMC3087556; doi:10.1186/1471-2148-10-91)
Supplement: Additional file 4 — Alignment of the Rp49 sequences from Florianópolis and Itaparica. Alignment of the DNA sequences from the Rp49 gene fragment from Florianópolis and Itaparica. The translated amino acid sequence is shown above the alignment and the intron is highlighted in grey. Dots represent identity and dashed represent gaps. The asterisks in the bottom line represent identity of all sequences. Flo: individuals from Florianópolis and Bah: individuals from Itaparica. [file 1471-2148-10-91-S4.DOC]

00000000000000000000000000000000000000000000000000000000000000000000000000000000000000000000000000011111111111111111111111111111111111111111111111111111111111111111111111111111111111111111111111111112222222222222222222222222222222222222222222222222222222222222222222222

00000000011111111112222222222333333333344444444445555555555566666666677777777778888888888999999999900000000001111111111222222222233333333334444444444555555555566666666667777777777888888888899999999990000000000111111111122222222223333333333444444444455555555556666666666

12345678901234567890123456789012345678901234567890123456789012345678901234567890123456789012345678901234567890123456789012345678901234567890123456789012345678901234567890123456789012345678901234567890123456789012345678901234567890123456789012345678901234567890123456789

I R H Q S D R Y D K L A P A W R R P K G I D N R V R R R F K G Q Y L M P N I G Y G S N K R T R H M L P C G F K K F L V H N V R

Bah10a TATCCGCCACCAGTCGGATCGCTATGACAAGCTTGCAGTAAGTGTTT--C-CGTTAAGGTTTGGGGTCTGGCGAAGTTTGTTGACCGTTTGCTTCGCATTCCTATTCCTCCTGCAGCCTGCATGGCGTCGGCCGAAAGGTATCGACAACCGGGTGCGTCGTCGCTTCAAGGGACAGTACCTGATGCCCAACATCGGTTACGGCTCGAACAAGCGCACACGCCATATGCTGCCGTGCGGATTCAAGAAGTTCCTCGTCCACAACGTGCGC

Bah10b ...............................................--.-..........................................................................................................................................................................................................................

Bah01a ...............................................--.C....................................A.............T....T..................................................................................................................................................................

Bah01b ...............................................--.-..........................................................................................................................................................................................................................

Bah03a ...............................................--.-..........................................................................................................................................................................................................................

Bah03b ...............................................--.-..........................................................................................................................................................................................................................

Bah07a ...............................................--.C....................T.............................T.......................................................................................................................................................................

Bah07b ...............................................--.-..........................................................................................................................................................................................................................

Bah08a ...............................................--.C....................T.............................T.......................................................................................................................................................................

Bah08b ...............................................--.C....................T.......A.....................T.......................................................................................................................................................................

Bah09a ...............................................--.-..................................................T.......................................................................................................................................................................

Bah09b ...............................................--.C..................................................T......................................................................................................................................................................T

Bah13a ...............................................--.C....................T.......A.....................T.......................................................................................................................................................................

Bah13b ...............................................--.-..........................................................................................................................................................................................................................

Bah14a ...............................................--.C....................T.............G...............T..........A............................................................................................................................................................

Bah14b ...............................................--.C................T...T.............................T.......................................................................................................................................................................

Bah15a ...............................................--.T....................T.............................T.......................................................................................................................................................................

Bah15b ...............................................--.C................T...T.............................T.......................................................................................................................................................................

Bah16a ...............................................--.C................T...T.............................T.......................................................................................................................................................................

Bah16b ...............................................--.C................T...T.............................T.......................................................................................................................................................................

Bah17a ...............................................--.C..................................................T.......................................................................................................................................................................

Bah17b ...............................................--.C..................................................T.......................................................................................................................................................................

Bah18a ...............................................--.-..................................................T.......................................................................................................................................................................

Bah18b ...............................................--.C..................................................T.......................................................................................................................................................................

Flo01a ...T..........................................CGC.G.A..GG..C...A.A.....................G......T......T......C.T.....................................T....................................................................C..T.................C..............................

Flo01b ...T..........................................CGC.G.A..G..AC...A.......................G......T..------.....C.T.....................................T....................................................................C..T.................C..............................

Flo03a ...T..........................................CGC.G.A..G...C...A.......................G......T......T......C.T.....................................T....................................................................C..T.................C..............................

Flo03b ...T..........................................CGC.G.A..G...C...A.......................G......T......T......C.T.....................................T....................................................................C..T.................C..............................

Flo04a ...T..........................................CGC.G.A..GG..C...A.A.....................G......T......T......C.T.....................................T....................................................................C..T.................C..............................

Flo04b ...T..........................................CGC.G.A..GG..C...A.A.....................G......T......T......C.T.....................................T....................................................................C..T.................C..............................

Flo05a ...T..........................................CGC.G.A..GG..C...A.A.....................G......T......T......C.T.....................................T....................................................................C..T.................C..............................

Flo05b ...T..........................................CGC.G.A..G...C...A.......................G......T......T......C.T.....................................T........................................................A...........C..T.................C..............................

Flo06a ...T..........................................CGC.G.A..G...C...A.......................G......T..------.....C.T.....................................T........................................................A...........C..T.................C..............................

Flo06b ...T..........................................CGC.G.A..G...C...A.......................G......T..------.....C.T.....................................T........................................................A...........C..T.................C..............................

Flo08a ...T..........................................CGC.G.A..G...C...A.......................G......T......T......C.T.....................................T........................................................A...........C..T.................C..............................

Flo08b ...T..........................................CGC.G.A..G...C...A.......................G......T..G...T......C.T.....................................T....................................................................C..T.................C..............................

Flo09a ..............................................CGC.G.A..G...CC..A.......................G......T......T......C.T.....................................T....................................................................C..T...........T.....C..............................

Flo09b ...T..........................................CGC.G.A..GG..C...A.......................G......T......T......C.T.....................................T....................................................................C..T...........T.....C..............................

Flo10a ..............................................CGC.GAA..G...C...A.......................G......T......T......C.T.....................................T....................................................................C..T.................C..............................

Flo10b ..............................................CGC.GAA..G...C...A.......................G......T......T......C.T.....................................T....................................................................C..T.................C..............................

Flo12a ...T..........................................CGC.G.A..G..AC...A.......................G......T..------.....C.T.....................................T....................................................................C..T.................C..............................

Flo12b ..............................................CGT.G.A..G...CG..A.......................G......T..------.....C.T.....................................T....................................................................C..T.................C..............................

Flo15a ...T..........................................CGC.G.A..GG..C...A.A.....................G......T......T......C.T.....................................T....................................................................C..T.................C..............................

Flo15b ...T..........................................CGC.G.A..G...C...A.......................G......T......T......CAT.....................................T....................................................................C..T.................C..............................

Flo16a ...T..........................................CGC.G.A..G...C...A.......................G......T..------.....C.T.....................................T....................................................................C..T.................C..............................

Flo16b ...T..........................................CGC.G.A..G...C...A.......................G......T..------.....C.T.....................................T....................................................................C..T.................C..............................

Flo17a ...T..........................................CGC.G.A..G..AC...A.......................G......T..------.....C.T.....................................T....................................................................C..T.................C..............................

Flo17b ...T..........................................CGC.G.A..GG..C...A.......................G......T......T......C.T.....................................T....................................................................C..T.................C..............................

*** ****************************************** * ** * ** * * *** ******* ***** * ****** ** *** * * *********************************** ******************************************************** *********** ** *********** ***** *****************************
