# Supplementary material for: Estimation of divergence time between two sibling species of the Anopheles (Kerteszia) cruzii complex using a multilocus approach
Source: BMC Evol Biol. 2010 Mar 31;10:91. doi: 10.1186/1471-2148-10-91 (PMC3087556; doi:10.1186/1471-2148-10-91)
Supplement: Additional file 5 — Alignment of the RpS2 sequences from Florianópolis and Itaparica. Alignment of the DNA sequences from the RpS2 gene fragment from Florianópolis and Itaparica. The translated amino acid sequence is shown above the alignment and the intron is highlighted in grey. Dots represent identity and dashed represent gaps. The asterisks in the bottom line represent identity of all sequences. Flo: individuals from Florianópolis and Bah: individuals from Itaparica. [file 1471-2148-10-91-S5.DOC]

000000000000000000000000000000000000000000000000000000000000000000000000000000000000000000000000000111111111111111111111111111111111111111111111111111111111111111111111111111111111111111111111111111122222222222222222222222222222222222222222222222222222222222222222222222

000000000111111111122222222223333333333444444444455555555555666666666777777777788888888889999999999000000000011111111112222222222333333333344444444445555555555666666666677777777778888888888999999999900000000001111111111222222222233333333334444444444555555555566666666667

123456789012345678901234567890123456789012345678901234567890123456789012345678901234567890123456789012345678901234567890123456789012345678901234567890123456789012345678901234567890123456789012345678901234567890123456789012345678901234567890123456789012345678901234567890

G K P H T V P C K V S G

Bah01a TCGGTAAACCACATACCGTACCGTGCAAGGTAAGTCATGATGCTGTTTTTTGGCCTAACAGACTAAGCGAACCGATTGGCGTGGCTTCCAAATGATGAGAAAAATTCAAAACTTATGCGGTTTTCACGGATAGGTGGCTGCATACCACTCGACTGACGTTCTATAAGCGTCTGATGGAACCACTCCTCAAAACGCC-AATGTTTCGCTTGAACCGTGATTAGCTCCGATTTCTGTAATGGGTTATGTTATTTTATTCGTAGGTCAGTGGC

Bah01b ....................................................................................................................................................................................................-.........................T..........C................................C...

Bah02a .................................A..................................................................................................................................................................-.........................T..........C....................................

Bah02b ....................................................................................................................................................................................................-.........................................................................

Bah03a ...........................................................................................................................................................................................GG.......-.........................T..........C................................C...

Bah03b ...........................................................................................................................................................................................GG.......-.........................T..........C................................C...

Bah04a ....C...............................................................................................................................................................................................-.........................T..........C....................................

Bah04b ...........................................................................................................................................................................................GG.......-.........................T..........C................................C...

Bah06a ....................................................................A.......-.......................................................................................................................-.........................T..........C....................................

Bah06b ....C...............................................................................................................................................................................................-.........................T..........C....................................

Bah07a ....C...............................................................................................................................................................................................-.........................T..........C....................................

Bah07b ....................................................................................................................................................................................................-.........................T..........C................................C...

Bah08a ....C...............................................................................................................................................................................................-.........................T..........C................................C...

Bah08b ....................................................................................................................................................................................................-....................................C....................................

Bah09a ...........................................................................................................................................................................................GG.......-.........................T..........C................................C...

Bah09b ....................................................................................................................................................................................................-.........................T..........C................................C...

Bah10a ....................................................................................................................................................................................................-.........................T..........C................................C...

Bah10b ....................................................................................................................................................................................................-.........................T..........C................................C...

Bah11a ...........................................................................................................................................................................................GG.......-.........................T..........C................................C...

Bah11b ....................................................................................................................................................................................................-.........................T..........C................................C...

Bah14a ....C...............................................................................................................................................................................................-.........................T..........C....................................

Bah14b ....................................................................................................................................................................................................-.........................T..........C................................C...

Bah15a ....................................................................................................................................................................................................-.........................................................................

Bah15b .................................A..................................................................................................................................................................-.........................T..........C....................................

Flo01a .............C....................C...........A---.........GA.......................T.....................................................A..........G........G.T..............................G....A..C......T......T.C......T......C...C.............A.....TAC.A........C...

Flo01b .............C....................C...........A---.........GA.......................T.....................................................A..........G........G.T..............................G....A..C......T......T.C......T......C...C.............A.....TAC.A........C...

Flo03a .............C....................C............---.........GA.................A.....T.....................................................A..........G........G.T......................G.......G....A..C.............T.C.T....T......C...C.............A.....TAC.A........C...

Flo03b .............C....................C............---.........GA.......................T.A...................................................A..........G........G.T..............................G....G..C.............T.C......T......C...C.............A.....TAC.A........C...

Flo04a .............C....................C............---.........GA.................A.....T.....................................................A..........G........G.T......................G.......G....A..C.............T.C.T....T......C...C.............A.....TAC.A........C...

Flo04b .............C....................C............---.........GA.................A.....T.............A.......................................A..........G........G.T..............TT..............G....G..C.............T.C......T......C...CA............A.....TAC.A........C...

Flo05a .............C....................C...........A---.........GA.......................T.....................................................A..........G........G.T..............................G....A..C......T......T.C......T......C...C.............A.....TAC.A........C...

Flo05b .............C....................C............---.........GA.................A.....T.............A.......................................A..........G........G.T..............................G....G..C.............T.C......T......C...CA............A.....TAC.A........C...

Flo06a .............C....................C............---.........GA.................A.....T.....................................................A..........G........G.T..............................G....G..C.............T.C......T......C...C.............A.....TAC.A........C...

Flo06b .............C....................C............---......G..GA.......................T.....................................................A..........G........G.T..............................G....A..C.............T.C......T......C...C...........-----...TAC.A........C...

Flo07a .............C....................C...........A---.........GA.......................T.....................................................A..........G........G.T..............................G....A..C......T......T.C......T......C...C.............A.....TAC.A........C...

Flo07b .............C....................C............---.........GA.........T.......A.....T.....................................................A..........G........G.T......................G.......G....A..C.............T.C.T....T......C...C.............A.....TAC.A........C...

Flo09a .............C....................C............---.........GA.........T.......A.....T.....................................................A..........G........G.T......................G.......G....A..C.............T.C.T....T......C...C.............A.....TAC.A........C...

Flo09b .............C....................C............---.........GA.........T.......A.....T.............A.......................................A..........G........G.T..............................G....G..C.............T.C......T......C...CA............A.....TAC.A........C...

Flo11a .............C....................C............---.........GA.................A.....T.............A.......................................A..........G........G.T..............................G....G..C.............T.C......T......C...CA............A.....TAC.A........C...

Flo11b .............C....................C............---.........GA.......................T.A...................................................A..........G........G.T..............................G....G..C.............T.C......T......C...C.............A.....TAC.A........C...

Flo12a .............C....................C............---.........GA.................A.....T.....................................................A..........G........G.T......................G.......G....A..C.............T.C.T....T......C...C.............A.....TAC.A........C...

Flo12b .............C.................................---..C......GA.....................A.T.....................................................A..........G........G.T..............................G....A..C.............T.C......T......C...C.............A.....CAC.A........C...

Flo13a .............C....................C............---.........GA.................A.....T.....................................................A..........G........G.T..............................G....G..C.............T.C......T......C...C.............A.....TAC.A........C...

Flo13b .............C....................C............---......G..GA.......................T.....................................................A..........G........G.T..............................G....A..C.............T.C......T......C...C.............A.....TAC.A........C...

Flo16a .............C.................................---..C......GA.....................A.T.....................................................A..........G........G.T..............................G....A..C.............T.C......T......C...C.............A.....CAC.A........C...

Flo16b .............C....................C............---.........GA.................A.....T.....................................................A..........G........G.T......................A.......G....A..C.............T.C.T....T......C...C.............A.....TAC.A........C...

Flo18a .............C....................C............---.........GA.................A.....T.....................................................A..........G........G.T......................A.......G....A..C.............T.C.T....T......C...C.............A.....TAC.A........C...

Flo18b .............C....................C............---.........GA.........T.......A.....T.....................................................A..........G........G.T......................G.......G....A..C.............T.C.T....T......C...C.............A.....TAC.A........C...

**** ******** ******************* *********** ** *** ** ******* * ***** * *** * * *********** *************************************** ********** ******** * ************** ****** *** ** **** ** ****** ****** * * **** ****** *** ********** *** * ******** ***
