# Supplementary material for: Estimation of divergence time between two sibling species of the Anopheles (Kerteszia) cruzii complex using a multilocus approach
Source: BMC Evol Biol. 2010 Mar 31;10:91. doi: 10.1186/1471-2148-10-91 (PMC3087556; doi:10.1186/1471-2148-10-91)
Supplement: Additional file 6 — Alignment of the RpS29 sequences from Florianópolis and Itaparica. Alignment of the DNA sequences from the RpS29 gene fragment from Florianópolis and Itaparica. The translated amino acid sequence is shown above the alignment and the intron is highlighted in grey. Dots represent identity and dashed represent gaps. The asterisks in the bottom line represent identity of all sequences. Flo: individuals from Florianópolis and Bah: individuals from Itaparica. [file 1471-2148-10-91-S6.DOC]

0000000000000000000000000000000000000000000000000000000000000000000000000000000000000000000000000001111111111111111111111111111111111111111111111111111111111111111111111111111111111111111111111111111222222222222222222222222222222222222222222222222222222222222222222222222222

0000000001111111111222222222233333333334444444444555555555556666666667777777777888888888899999999990000000000111111111122222222223333333333444444444455555555556666666666777777777788888888889999999999000000000011111111112222222222333333333344444444445555555555666666666677777

1234567890123456789012345678901234567890123456789012345678901234567890123456789012345678901234567890123456789012345678901234567890123456789012345678901234567890123456789012345678901234567890123456789012345678901234567890123456789012345678901234567890123456789012345678901234

G Q G S R F C R A C S N N H G M I R K Y G L N I C R Q C F R E Y A

Bah08b CGGACAGGGATCGCGATTCTGGTGAGTTTCGTACCGAGCTGACGAGTTGTGCGGGTAACTTGTGAGGAAGAAAGCGGCCGAACGAATCGGCTGTTTGGCACGTGTACTGTGAGCGTAGCCTCGCCATCTTGAAGCCACTGGTATCGTTTACGTTGGGCGGCAACAACCCATTCATATCCGTTTTCTCGTTTTAGCCGTGCCTGCTCCAACAATCACGGAATGATCCGAAAGTACGGACTCAACATTTGCCGACAGTGCTTCCGCGAGTACGCGA

Bah08a ...................................A.......................................................................................................................................................A........................C.............................................................

Bah01a ...................................A.................................................G..............................................................G......................................A........................C.............................................................

Bah01b ...................................A................................................................................................................................................................................C.............................................................

Bah07a ...................................A................................................................................................................................................................................C.............................................................

Bah07b ...................................A................................................................................................................................................................................C.............................................................

Bah09a ...................................A.........................................T......................................T.............................................................................................................................................................

Bah09b ...................................A..............................................................................................................................................................................................................................................

Bah10a ...................................A..............................................................................................................................................................................................................................................

Bah10b ...................................A..............................................................................................................................................................................................................................................

Bah13a ...................................A.......................................................................................................................................................A........................C.............................................................

Bah13b ...................................A.......................................................................................................................................................A........................C.............................................................

Bah24a ...................................A...................C..........................................................................................................................................................................................................................

Bah24b ...................................A.......................................................................................................................................................A........................C......................................C......................

Bah27a ...................................A.......................................................................................................................................................A........................C.............................................................

Bah27b ...................................A.......................................................................................................................................................A........................C.............................................................

Bah28a ...................................A........................................................................T..............................................................................A........................C.............................................................

Bah28b ...................................A.................................................G..............................................................G......................................A........................C.............................................................

Bah29a ...................................A....................................................................................................................................A..................A........................C.............................................................

Bah29b ...................................A.......................................................................................................................................................A........................C.............................................................

Bah30a ...................................A........................................................................T..............................................................................A........................C.............................................................

Bah30b ...................................A.............................................................................................................................T.........C...............A........................C.............................................................

Bah31a ...................................A.......................................................................................................................................................A........................C.............................................................

Bah31b ...................................A...........G.....................A.......A....A......CGCC.....................................................................................................................................................................................

Flo01a ...................................A..A........G......A..GT....A...C........A............CGCC...................C.A....................T..CTT......G............T.....T....................A......T.................C.......................T.....................................

Flo01b .........G.........................A..A........G......A..GT....A...C........A............CGCC...................C.A....................TG----...................T.....T....................A......T.................C.......................T.....................................

Flo03a ...................................A..A........G......A..GT....A...C........A............CGCC...................C.A...................ATG----...................T.....T....................A......T.................C.......................T.....................................

Flo03b .........G.........................A..A........G......A..GTG...A...C........A............CGCC...................C.A....................TG----........G..........T.....T....................A......T.................C.......................T.....................................

Flo04a ...................................A..A........G......A..GT....A...C........A............CGCC.................T.C.A....................T..CTT......G............T.....T....................A......T.................C.......................T.....................................

Flo04b .........G.........................A..A........G......A..GT....A...C........A............CGCC...................C.A....................TG----...................T.....T....................A......T.................C.......................T.....................................

Flo06a .........G.........................A..A........G......A..GT....A...C........A............CGCC...................C.A....................T..CTT......G............T.....T....................A......T.................C.......................T.....................................

Flo06b .........G.........................A..A........G......A..GT....A...C........A............CGCC...................C.A....................TG----...................T.....T....................A......T.................C.......................T.....................................

Flo07a ...................................A..A........G......A..GT....A...C........A............CGCC.................T.C.A....................T..CTT......G............T.....T..................C.A......T.................C.......................T.....................................

Flo07b .........G.........................A..A........G......A...A....A...C........A...G........CGCC...................C.A....................TG----...................T.....TT...................A......T.................C.......................T.....................................

Flo09a .........G.........................A..A........G......A..GT....A...C........A............CGCC...................C.A....................TG----...................T.....T....................A......T.................C.......................T.....................................

Flo09b ...................................A..A........G......A..GT....A...C........A............CGCC.................T.C.A....................TG----...................T.....T....................A......T.................C.......................T.....................................

Flo11a ...................................A..A........G......A..GT....A...C........A............CGCC...................C.A....................TG----...................T.....T....................A......T.................C.......................T.....................................

Flo11b ...................................A..A........G......A..GT....A...C........A............CGCC.................T.C.A....................T..CTT......G............T.....T....................A......T.................C.......................T.....................................

Flo12a ...................................A..A........G......A..GT....A...C........A............CGCC.................T.C.A....................T.-------------.....A....TG....T----........T.......A........................C.......................T.....................................

Flo12b ...................................A..A........G......A..GT....A...C........A............CGCC.................T.C.A....................T..CTT......G............T.....T....................A......T.................C.......................T.....................................

Flo13a ...................................A..A........G......A..GT....A...C........A............CGCC...................C.A....................T..CTT......G............T.....T....................A......T.................C.......................T.....................................

Flo13b .........G.........................A..A........G......A..GTG...A...C........A............CGCC...................C.A....................TG----...................T.....T....................A......T.................C.......................T.....................................

Flo15a ...................................A..A........G......A..GT....A...C........A............CGCC...................C.A....................TG----...................T.....T....................A......T.................C.......................T.....................................

Flo15b ...................................A..A........G......A..GT....A...C........A............CGCC...................C.A....................T..CTT......G............T.....T....................A......T.................C.......................T.....................................

Flo16a .........G.........................A..A........G......A..GT....A...C........A............CGCC...................C.A....................T..CTT......G............T.....T....................A......T.................C.......................T.....................................

Flo16b .........G.........................A..A........G......A..GT....A...C........A............CGCC...................C.A....................TG----...................T.....T....................A......T.................C.......................T.....................................

Flo17a .........G.........................A..A........G......A..GT....A...C........A............CGCC...................C.A....................TG----...................T.....T....................A......T.................C.......................T.....................................

Flo17b .........G.........................A..A........G......A..GT....A...C........A............CGCC...................C.A....................TG----...................T.....T....................A......T.................C.......................T.....................................

********* ************************* ** ******** ****** * *** *** * ****** ** * ** *** *************** * * * * ***************** ***** **** **** ******* ***** * ****** ***************** *********************** ************** **********************
