# Supplementary material for: Estimation of divergence time between two sibling species of the Anopheles (Kerteszia) cruzii complex using a multilocus approach
Source: BMC Evol Biol. 2010 Mar 31;10:91. doi: 10.1186/1471-2148-10-91 (PMC3087556; doi:10.1186/1471-2148-10-91)
Supplement: Additional file 7 — Summarized features of the marginal histograms for each parameter. Values of the six parameters that span the prior distribution are presented for each of the four runs with different seed numbers (a, b, c and d). Population size parameter for Itaparica, Florianópolis and ancestral populations (θ1, θ2, θA); Time of population splitting parameter (t); Migration rate estimate from Florianópolis to Itaparica population (m1) and from Itaparica to Florianópolis population (m2); Minbin and Maxbin, the midpoint values of the lowest and the highest bin, respectively; HiPt, the value of the bin with the highest count; HiSmth, the value of the bin with the highest count, after the counts have been smoothed by taking a running average of 9 points centered on each bin; 95Lo and 95Hi, the estimated points to which 2.5% of the total area lies to the left and to the right, respectively; HPD90Lo and HPD90Hi, the lower and upper bounds of the estimated 90% highest posterior density (HPD) interval, respectively. [file 1471-2148-10-91-S7.DOC]

|  |  | Minbin | Maxbin | HiPt | HiSmth | Mean | 95Lo | 95Hi | HPD90Lo | HPD90Hi |
| --- | --- | --- | --- | --- | --- | --- | --- | --- | --- | --- |
| θ_1_ | **A** | 0.6770 | 5.5448 | 2.0120 | 2.0057 | 2.0624 | 1.3886 | 3.0007 | 1.4200 | 2.7425 |
|  | **B** | 0.6644 | 5.7211 | 1.9805 | 1.9931 | 2.0624 | 1.3886 | 3.0007 | 1.4200 | 2.7425 |
|  | **C** | 0.7210 | 5.6518 | 1.9868 | 1.9805 | 2.0624 | 1.3886 | 3.0007 | 1.4200 | 2.7425 |
|  | **D** | 0.7210 | 6.2312 | 1.9994 | 1.9931 | 2.0624 | 1.3886 | 3.0007 | 1.4200 | 2.7425 |
| θ_2_ | **A** | 1.0659 | 7.2149 | 2.7815 | 2.7475 | 2.8410 | 1.9662 | 4.0555 | 2.0086 | 3.7242 |
|  | **B** | 0.9385 | 7.6141 | 2.7815 | 2.7730 | 2.8410 | 1.9662 | 4.0555 | 2.0086 | 3.7242 |
|  | **C** | 1.0064 | 7.9114 | 2.7815 | 2.7730 | 2.8410 | 1.9662 | 4.0555 | 2.0086 | 3.7242 |
|  | **D** | 0.9810 | 7.5801 | 2.7900 | 2.7475 | 2.8410 | 1.9662 | 4.0555 | 2.0086 | 3.7242 |
| θ_A_ | **A** | 0.0099 | 19.8074 | 0.2081 | 0.1883 | 3.9734 | 0.1883 | 16.4583 | 0.0099 | 11.3652 |
|  | **B** | 0.0099 | 19.8074 | 0.0495 | 0.1288 | 3.9139 | 0.1684 | 16.4781 | 0.0099 | 11.3454 |
|  | **C** | 0.0099 | 19.8074 | 0.0099 | 0.0099 | 3.9932 | 0.1684 | 16.5178 | 0.0099 | 11.4049 |
|  | **D** | 0.0099 | 19.8074 | 0.0099 | 0.0099 | 3.9932 | 0.1883 | 16.5178 | 0.0099 | 11.4247 |
| *t* | **A** | 1.1300 | 19.9900 | 5.6700 | 5.6500 | 5.7300 | 2.8900 | 12.9100 | 2.7300 | 8.4900 |
|  | **B** | 1.2300 | 19.9900 | 5.6700 | 5.6900 | 5.7700 | 2.9500 | 13.3900 | 2.7500 | 8.5900 |
|  | **C** | 1.0900 | 19.9900 | 5.7900 | 5.7700 | 5.7500 | 2.8900 | 13.5500 | 2.6900 | 8.5700 |
|  | **D** | 1.2300 | 19.9900 | 5.6700 | 5.7100 | 5.7300 | 2.8900 | 13.4300 | 2.6700 | 8.5300 |
| *m_1_* | **A** | 0.0001 | 0.0999 | 0.0003 | 0.0003 | 0.0201 | 0.0008 | 0.0854 | 0.0001 | 0.0617 |
|  | **B** | 0.0001 | 0.0999 | 0.0001 | 0.0001 | 0.0203 | 0.0008 | 0.0858 | 0.0001 | 0.0622 |
|  | **C** | 0.0001 | 0.0999 | 0.0001 | 0.0001 | 0.0202 | 0.0008 | 0.0857 | 0.0001 | 0.0621 |
|  | **D** | 0.0001 | 0.0999 | 0.0004 | 0.0003 | 0.0202 | 0.0008 | 0.0857 | 0.0001 | 0.0620 |
| *m_2_* | **A** | 0.0001 | 0.0999 | 0.0002 | 0.0002 | 0.0154 | 0.0006 | 0.0764 | 0.0001 | 0.0506 |
|  | **B** | 0.0001 | 0.0999 | 0.0001 | 0.0001 | 0.0155 | 0.0006 | 0.0765 | 0.0001 | 0.0507 |
|  | **C** | 0.0001 | 0.0999 | 0.0001 | 0.0001 | 0.0155 | 0.0006 | 0.0767 | 0.0001 | 0.0508 |
|  | **D** | 0.0001 | 0.0999 | 0.0001 | 0.0001 | 0.0154 | 0.0006 | 0.0765 | 0.0001 | 0.0507 |
