# Supplementary figures and images for: Estimation of divergence time between two sibling species of the Anopheles (Kerteszia) cruzii complex using a multilocus approach
Source: BMC Evol Biol. 2010 Mar 31;10:91. doi: 10.1186/1471-2148-10-91 (PMC3087556; doi:10.1186/1471-2148-10-91)

## Slide 1
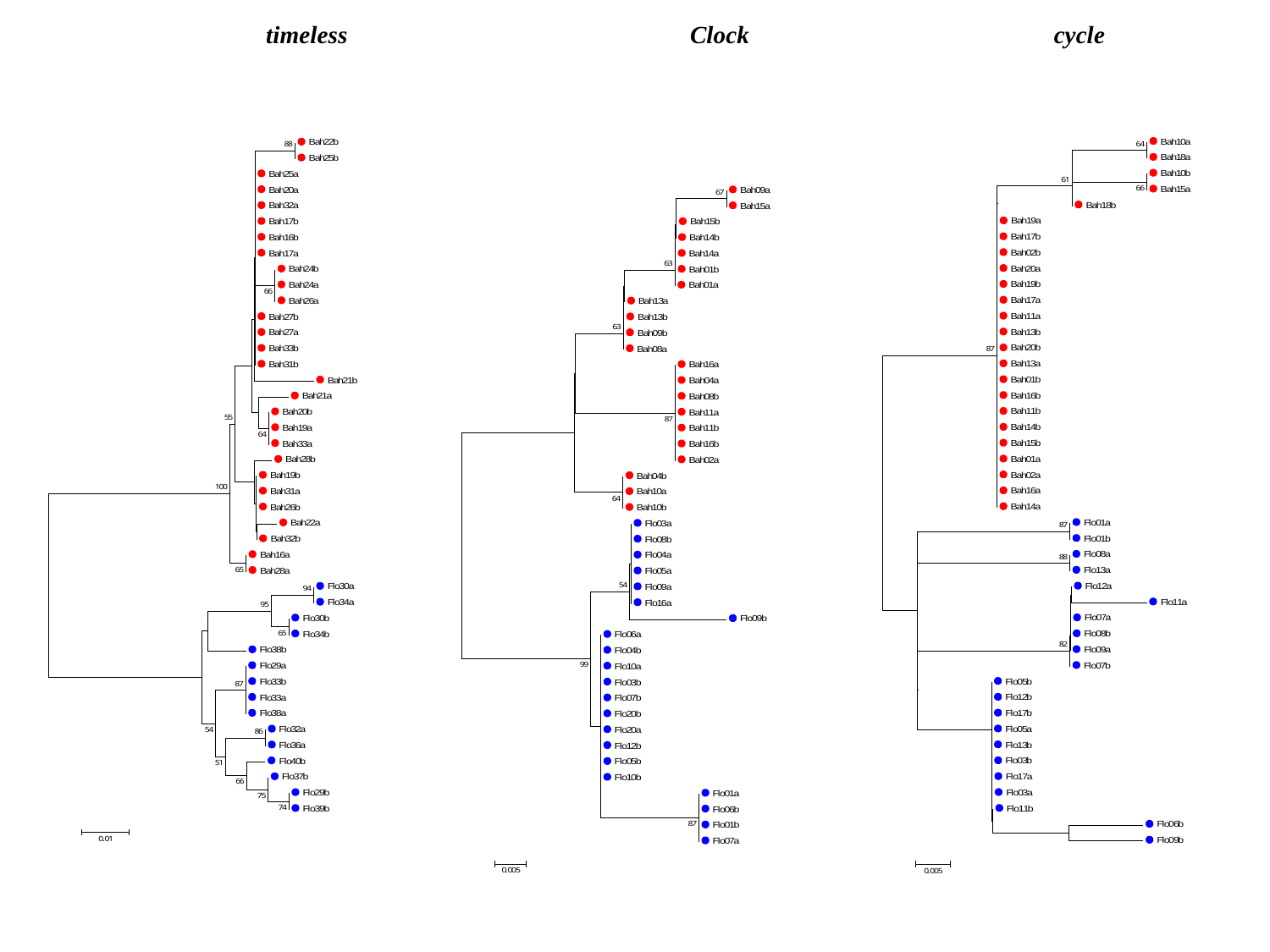

timeless
Clock
cycle

Supplement: Additional file 8 — Neighbor-joining trees of NR blocks of clock genes. The trees were estimated using the neighbor-joining method with 1,000 bootstrap replicates. The Jukes and Cantor distance was used for the Clock gene and Kimura 2-parameter distance for the others. [file 1471-2148-10-91-S8.PPT]

## Slide 1
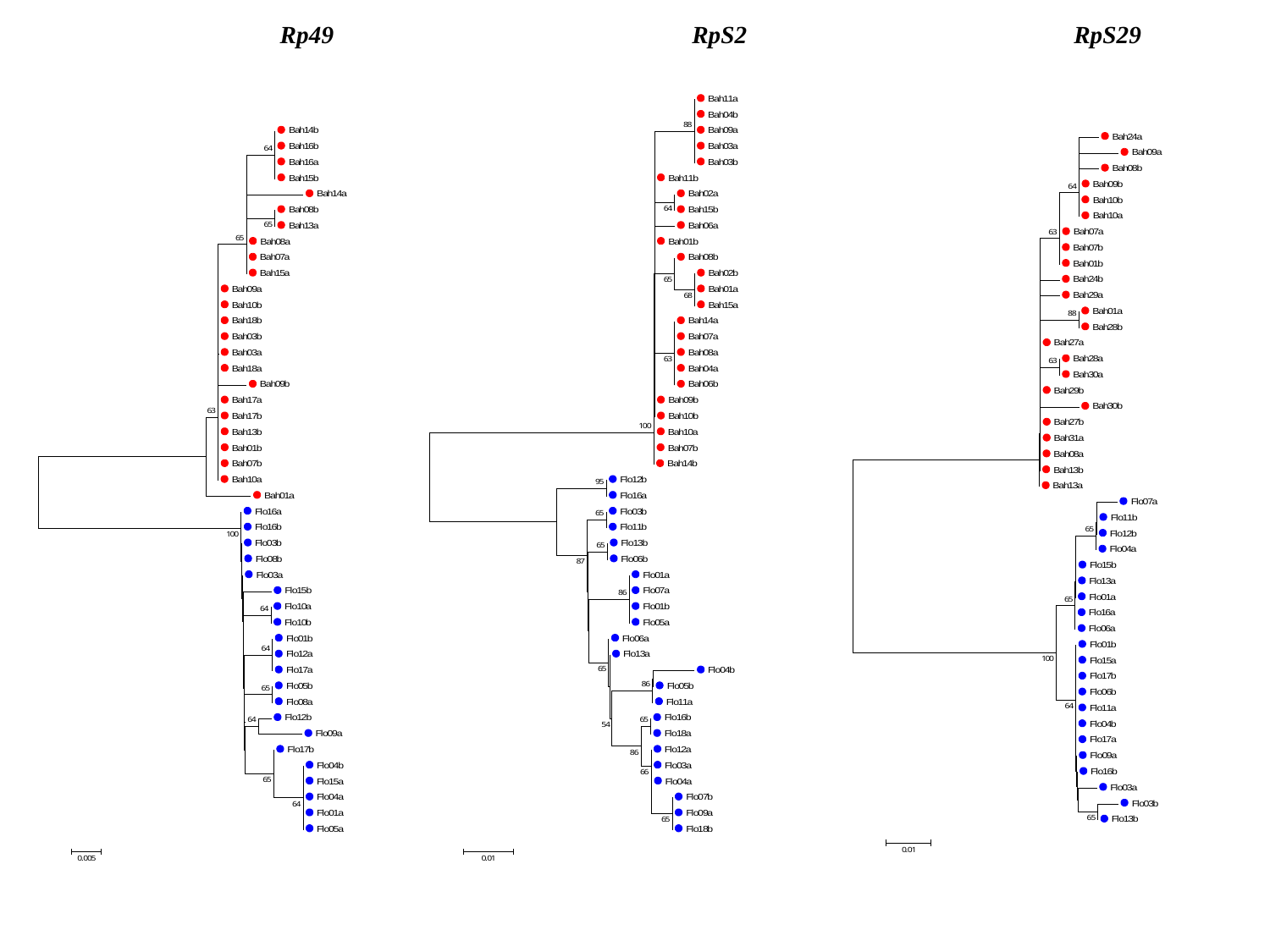

Rp49
RpS2
RpS29

Supplement: Additional file 9 — Neighbor-joining trees of NR blocks of ribosomal protein genes. The trees were estimated using the neighbor-joining method with 1,000 bootstrap replicates and Kimura 2-parameter distance. [file 1471-2148-10-91-S9.PPT]
